# Plant Mitogen-Activated Protein Kinase Cascades in Environmental Stresses

**DOI:** 10.3390/ijms22041543

**Published:** 2021-02-03

**Authors:** Li Lin, Jian Wu, Mingyi Jiang, Youping Wang

**Affiliations:** 1 Key Laboratory of Plant Functional Genomics of the Ministry of Education, Yangzhou University, Yangzhou 225000, China; m160780@yzu.edu.cn; 2 Jiangsu Key Laboratory of Crop Genomics and Molecular Breeding, Yangzhou University, Yangzhou 225000, China; 3 College of Life Sciences and National Key Laboratory of Crop Genetics and Germplasm Enhancement, Nanjing Agricultural University, Nanjing 210095, China; myjiang@njau.edu.cn

**Keywords:** MAPK cascade, abiotic stress, biotic stress, signal transduction

## Abstract

Due to global warming and population growth, plants need to rescue themselves, especially in unfavorable environments, to fulfill food requirements because they are sessile organisms. Stress signal sensing is a crucial step that determines the appropriate response which, ultimately, determines the survival of plants. As important signaling modules in eukaryotes, plant mitogen-activated protein kinase (MAPK) cascades play a key role in regulating responses to the following four major environmental stresses: high salinity, drought, extreme temperature and insect and pathogen infections. MAPK cascades are involved in responses to these environmental stresses by regulating the expression of related genes, plant hormone production and crosstalk with other environmental stresses. In this review, we describe recent major studies investigating MAPK-mediated environmental stress responses. We also highlight the diverse function of MAPK cascades in environmental stress. These findings help us understand the regulatory network of MAPKs under environmental stress and provide another strategy to improve stress resistance in crops to ensure food security.

## 1. Introduction

Plants are confronted with multiple stresses during their lifetime. Environmental stresses are the most common stimuli affecting plant growth and development and eventually endanger crop production worldwide and threaten food security [1,2]. To date, almost 50% of crop yield reductions have been caused by environmental stresses [3,4]. In contrast to animals, when plants face stressful conditions, they become very passive. The only way to survive is to adapt to adverse conditions. Due to climate change exacerbation, the effect of environmental stresses is becoming increasingly adverse. How to improve stress tolerance in plants has become a hot topic for ensuring agricultural productivity [5]. During long-term cell–environment communication, plants have already evolved sophisticated and precise systems to adjust to changeable conditions [6,7]. Stimulus perception and reaction are closely related to the plant survival rate. Stimulus perception requires cell surface-located sensors/receptors to perceive diverse stresses and transduce these signals through several signaling pathways. As a key signaling module downstream of receptor-like protein kinases (RLKs), mitogen-activated protein kinase (MAPK) cascades act as a molecular switch in sensing upstream signaling and respond to environmental stresses, eventually determining the fate of plants under adverse conditions [6,8,9,10,11,12]. Therefore, understanding the function of plant MAPK cascades in environmental stresses will be beneficial for molecular breeding of novel stress-resistant crops.

In plants, the typical MAPK cascade comprises the following three family members: mitogen-activated protein kinase kinase kinases (MKKKs or MEKKs), mitogen-activated protein kinase kinases (MKKs or MEKs) and mitogen-activated protein kinases (MAPKs), which link extracellular stresses with the intercellular responses. In *Arabidopsis*, there are nearly 80 putative MKKKs, 10 MKKs and 20 MAPKs that form MAPK cascade components [13,14]. The MAPK cascade transduces and amplifies signals through sequential phosphorylation [12]. Activated MKKKs phosphorylate downstream MKKs, which, in turn, phosphorylate and activate MAPKs [15,16]. Activated MAPKs target specific downstream substrates, such as other kinases, enzymes and transcription factors [17,18,19]. Moreover, some research also investigated that the other protein kinases can involve in activating of MAPKs [20,21]. To date, most previous studies investigated the function of MPK3, MPK4 and MPK6 and discovered their upstream kinases and downstream targets. In this review, we aim to summarize current major developments in MAPK-mediated abiotic stress and biotic stress responses in plants and discuss the complex regulation networks of the MAPK cascade under diverse signaling pathways. Furthermore, we aim to provide some strategies to address MAPK-related environmental stress responses.

## 2. MAPK in Salt Stress

Due to incorrect irrigation, soil pollution and improper fertilizer application, at least 7% of the world’s area is affected by saline soil [22,23]. Salt stress has adverse effects on plant development and productivity and constrains crop production by 20% on irrigated land worldwide [24,25,26]. Hence, understanding how plants perceive high concentrations of salt and eventually adapt to salt stress is critical for breeding salt-tolerant crops [27,28]. Salt stress is complex and induces osmotic stress and oxidative stress [29,30]. MAPK cascade involvement in salt stress has been reported in *Arabidopsis*, rice, maize, cotton, etc. (Table 1). The MAPK cascade regulates plant tolerance to salt stress mainly by regulating the expression of salt-related genes, maintaining oxidative homeostasis and relieving osmotic stress [31].

**Figure 1:**
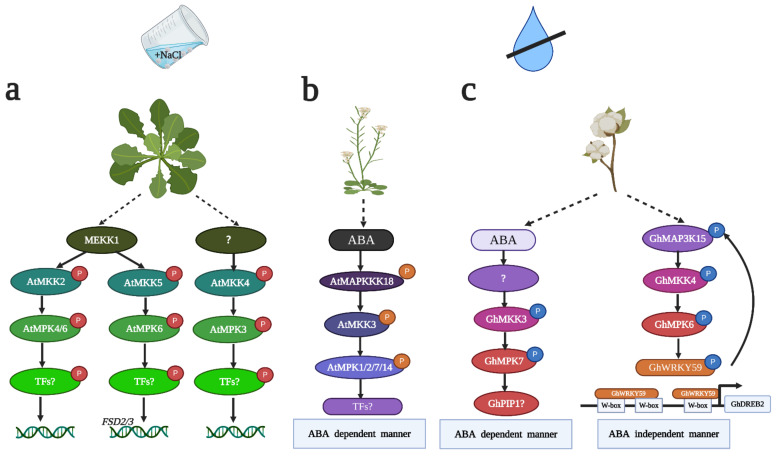
MAPK cascade in salt and drought stress. (**a**) The following three MAPK cascades can regulate salt stress in *Arabidopsis*: the AtMEKK1-AtMKK2-AtMPK4 cascade, the AtMEKK1-AtMKK5-AtMPK6 cascade and the AtMKK4-AtMPK3 cascade. The substrate of the AtMEKK1-AtMKK2-AtMPK4 and AtMKK4-AtMPK3 cascades is still unknown (marked as ?). The AtMEKK1-AtMKK5-AtMPK6 cascade confers tolerance to salt stress by regulating *AtFSD2/3* expression. *AtFSD2/3* are two major FSD-encoding genes in *Arabidopsis*. (**b**) The AtMAPKKK18-AtMKK3-AtMPK1/2/7/14 cascade can be activated by ABA after drought stress. The AtMAPKKK18-AtMKK3-AtMPK1/2/7/14 cascade positively regulates drought stress in an ABA-dependent manner. The substrate of AtMPK1/2/7/14 is unknown (marked as ?). (**c**) Two MAPK cascades are involved in drought stress in cotton. The GhMKK3-GhMPK7 cascade enhances drought tolerance in an ABA-dependent manner. Whether GhPIP1 is the substrate of GhMPK7 requires more experimental evidence (marked as ?). GhMAP3K15-GhMKK4-GhMPK6 positively regulates drought stress in an ABA-independent manner. The substrate of this cascade is GhWRKY59. GhWRKY59 can regulate *GhDREB2* expression by directly binding the W-box of *GhDREB2* promoters. This figure was created using BioRender (http://biorender.com/; accessed on 14 December 2020).

Upon salt stress, activated MAPK cascades trigger the altered transcription of salt-responsive genes [59]. The MAPK cascade becomes a link between salt stress sensors and target genes. However, evidence suggesting that the MAPK cascade directly regulates target genes is lacking. AtMEKK1-AtMKK2-AtMPK4/6 is the first complete MAPK signaling module identified in *Arabidopsis* that confers tolerance to salt stress [32] (Figure 1a). *AtMKK2*-overexpressing plants show an increased ratio of seed germination following NaCl treatment compared to *atmkk2* null mutant plants. AtMKK2 phosphorylates AtMPK4 and AtMPK6 in vivo and in vitro. A transcriptome analysis showed that 152 genes had changed expression in *AtMKK2*-overexpressing plants. These genes can be clustered into several types. Some genes are abiotic stress marker genes, and the other genes are involved in jasmonic acid (JA), ethylene (ET) and auxin signaling. However, to date, the target genes or transcriptional factors of AtMPK4/6 under salt stress are still unknown, and whether JA, ET and auxin signaling engages in crosstalk in AtMEKK1-AtMKK2-AtMPK4/6 module-mediated salt stress is even less clear. Recently, an ortholog of *AtMKK1* and *AtMPK4* in rice, *OsMKK1*-*OsMPK4*, also enhanced resistance to salt stress [60]. The expression levels of *OsDREB2A*, *OsDREB2B*, *OsNAC6* and *OsMYBS3* were decreased after NaCl treatment in *osmkk1* mutants, suggesting that OsMKK1-mediated salt tolerance relied on salt-responsive gene expression. However, the relationship between the OsMKK1-OsMPK4 cascade and these transcription factors is still unknown [60]. In addition, the function of MAPK in salt stress in maize and cotton has been elucidated. *ZmSIMK1*, *ZmMKK4*, *ZmMPK17*, *GhMPK2* and *GhMPK17* conferred tolerance to salt stress by regulating salt marker genes [39,40,43,52,55]. Overexpressing *ZmSIMK1* in *Arabidopsis* upregulated the expression levels of *AtRD29A* and *AtP5CS1* after NaCl treatment [40]. Overexpressing *ZmMKK4* in *Arabidopsis* increased the transcript levels of *AtP5CS2*, *AtRD29A*, *AtSTZ* and *AtDREB2A* after NaCl treatment [39]. Overexpressing *GhMPK17* in *Arabidopsis* increased the mRNA levels of *AtSOS2* after NaCl treatment [55]. Overexpressing *GhMPK2* in tobacco increased the expression levels of *NtDIN1*, *NtOsmotin* and *NtLEA5* after NaCl treatment [52]. Overexpressing *ZmMPK17* in tobacco resulted in higher transcript levels of *NtEDR10B* and *NtEDR10C* compared with those in the control plants after NaCl treatment [43]. In summary, these studies prove that the MAPK cascade responding to salt stress is closely related to the regulation of salt-responsive genes, but whether the MAPK cascade directly regulates salt-responsive genes needs to be further investigated.

Plants achieve tolerance to salinity by alleviating osmotic stress, which is known to induce cellular endogenous ABA concentrations [61]. It has been reported that MAPK signaling regulates salt stress in an ABA-dependent manner [62]. AtMAP3Kδ4 is an ABA-induced Raf-like MAP3K. Overexpressing *AtMAP3Kδ4* in *Arabidopsis* results in tolerance to NaCl treatment during germination, and overexpression plants exhibit lower sensitivity to ABA than control plants, suggesting that the mediation of salt tolerance by AtMAP3Kδ4 is correlated with ABA [33]. It has also been reported that GhMPK2 mediates resistance to salt stress by ABA-triggered osmotic stress [52]. Overexpressing *GhMPK2* in tobacco conferred tolerance to NaCl treatment during germination and growth. The mRNA of *GhMPK2* accumulated after ABA treatment; additionally, the overexpression plants showed higher germination and survival rates after ABA treatment, indicating that GhMPK2 positively regulates salt stress in an ABA-dependent manner [52]. Other studies have also shown that the MAPK cascade regulates ABA-dependent gene expression in response to salt stress. An MAPK cascade composed of AtMKK4-AtMPK3 plays a crucial role in salt stress in *Arabidopsis* [35]. *AtMKK4*-overexpressing and *atmkk4* mutant plants displayed opposite phenotypes under high salinity, and *atmkk4* mutant plants are more sensitive to salinity, whereas *AtMKK4*-overexpressing plants display salt tolerance. The transcription of *AtRD29A* and *AtNCED3* is decreased in *atmkk4* mutant plants but increased in *AtMKK4*-overexpressing plants. An in-gel kinase assay further indicated that AtMPK3 is downstream of AtMKK4 upon salinity stress [35]. Consistent with AtMPK3, GhMPK4 also regulates salt stress by altering ABA-dependent gene expression in *Arabidopsis*. However, the expression levels of *AtSOS2* and *AtRD29A* were remarkably reduced in *GhMPK4*-overexpressing transgenic plants under NaCl treatment. Therefore, GhMPK4 is a negative regulator in salt stress [54]. Cumulatively, ABA plays a crucial role in salt stress, but whether MAPK can phosphorylate ABA-dependent salt-related genes remains unclear.

As the second main source of stress in salt stress, oxidative stress can trigger the accumulation of reactive oxygen species (ROS), which have toxic effects on plants [26]. The MAPK cascade can regulate antioxidative response gene expression and increase antioxidative enzyme activities to detoxify ROS and sustain ROS homeostasis [25]. A complete MAPK cascade consisting of AtMEKK1-AtMKK5-AtMPK6 plays an essential role in the iron superoxide dismutase (FSD) signaling-mediated salt stress response in *Arabidopsis* [36,63] (Figure 1a). AtMKK5 can be activated after NaCl treatment, and overexpressing *AtMKK5* in *Arabidopsis* confers tolerance to salt stress [64]. *AtFSD2* and *AtFSD3* are two FSD-encoding genes that can be induced after NaCl treatment. However, the expression of *AtFSD2/3* was dramatically abolished in *AtMKK5*-RNAi plants but not in *AtMKK4*-RNAi plants or *atmkk2* mutants. The promoters of *AtFSD2* and *AtFSD3* are not activated in *mkk5* protoplasts, but the activation levels of the *FSD2* and *FSD3* promoters do not differ among wild-type (WT) plants, *AtMKK4*-RNAi plants and *atmkk2* mutant plants, suggesting that AtMKK5 is specifically involved in salt-induced FSD signaling in *Arabidopsis*. Yeast two-hybrid, in-gel kinase and transient assays in protoplasts prove that AtMEKK1 and AtMPK6 are involved in AtMKK5-mediated FSD signaling upon salt stress [36,64]. Although AtMEKK1-AtMKK5-AtMPK6 has been shown to participate in FSD signaling-induced salt stress, whether this MAPK cascade can directly regulate *FSD2* and *FSD3* requires additional molecular and genetic evidence. Other studies have also shown that the MAPK cascade regulates salt stress by changing antioxidative enzyme activities and cellular H_2_O_2_ contents. Overexpressing *ZmMKK4* in *Arabidopsis* conferred tolerance to salt stress by increasing POD (peroxidase) and CAT (catalase) activities [39]. Overexpressing *ZmMPK5* in tobacco increases the enzyme activities of CAT, POD, SOD (superoxide dismutase) and APX (ascorbate peroxidase) to confer salt stress resistance to transgenic plants [42]. Overexpressing *GhMPK17* in *Arabidopsis* resulted in less H_2_O_2_ accumulation than that observed in the control plants after NaCl treatment; thus, overexpression plants displayed resistance to salt stress [55]. However, overexpressing *GhRaf19* and *GhMKK5* in tobacco enhanced H_2_O_2_ production upon NaCl treatment. Thus, GhRaf19 and GhMKK5 negatively regulate salt stress by aggravating oxidative stress [46,50]. Accumulating data demonstrate that the relationship between oxidative stress and salt stress is antagonistic. However, numerous studies show that MAPK enhances salt tolerance by relieving oxidative stress based on DAB and NBT staining and SOD, POD, CAT and APX activity measurements. The mechanism by which MAPKs regulate these antioxidative enzymes is still unclear.

## 3. MAPK in Drought

Drought stress affecting food productivity has become a troublesome problem worldwide. Drought stress is a complex stress that causes multidimensional changes, such as physiological processes, molecular mechanisms and morphological adjustments [65,66,67,68]. Moreover, the effect caused by drought stress differs across developmental stages and plant species [69]. As a major signal transducer, the MAPK cascade plays a vital role in drought stress, generally by responding to ABA and regulating ROS production [20,21,70] (Table 1). Moreover, several WRKY transcription factors have been identified as substrates of the MAPK cascade in drought stress.

According to RNA-Seq analyses, numerous components of MAPK cascades have been reported to respond to drought in crops. In rice, the transcripts of *OsMKK4*, *OsMKK1*, *OsMPK8*, *OsMPK7*, *OsMPK5* and *OsMPK4* accumulate under drought stress [71,72,73,74,75,76]. In wheat, the expression levels of *TaRaf87*, *TaRaf105*, *TaRaf44*, *TaRaf72*, *TaRaf80*, *TaMKKK16*, *TaMKK1* and *TaMPK8* changed after drought stress [56,57]. In cotton, *GhRAF4*, *GhMEKK12*, *GhMEKK10*, *GhMEKK24* and *GhMEKK36* were induced after 8 days of drought [77], while the transcription levels of *GhMPK6*, *GhMPK9*, *GhMPK10*, *GhMPK12*, *GhMPK13*, *GhMPK19* and *GhMPK24* were strongly decreased after PEG6000 treatment [78]. In maize, *ZmMAPKKK56*, *ZmMAPKKK19*, *ZmMAPKKK18*, *ZmMKK10*-*2*, *ZmMPK3* and *ZmMPK15* were induced under drought conditions [38,41]. These findings highlight the importance of MAPKs in drought, but knowledge regarding their biological functions under drought stress is limited. Further studies should expand efforts to uncover their biological functions in drought stress.

Drought stress is often co-related with ABA and ROS accumulation. Thus, the mechanisms regulating drought stress can be classified as follows: ABA-mediated stomatal closure and ROS scavenging. Some studies have already proved the MAPK cascade to be involved in ABA signaling under drought conditions [11,34,48,52,79]. In *Arabidopsis*, the AtMAPKKK18-AtMAPKK3 pathway positively regulates drought stress via ABA-mediated stomatal closure (Figure 1b). *Atmapkkk18* mutant plants are more sensitive than WT plants to drought conditions, whereas *AtMAPKKK18* overexpression plants display tolerance to drought. Moreover, stomatal closure is faster in *AtMAPKKK18* overexpression plants but slower in *atmapkkk18* mutants, suggesting that a difference in stomatal closure is the reason for *AtMAPKKK18*-mediated drought tolerance. The *atmkk3* mutant also displays drought sensitivity, whereas *AtMKK3* overexpression plants display drought tolerance. When *AtMAPKKK18* is overexpressed in an *atmkk3* background, the plants exhibit suppressed drought tolerance, suggesting that AtMAPKKK18-AtMKK3-mediated drought tolerance is related to ABA [34]. Previous studies have already proven that AtMPK1/2/7/14 can interact with AtMKK3 [79], but the substrate of AtMKK3 in drought stress needs more experimental evidence. Furthermore, *GhMKK3*, which is an ortholog of *AtMKK3*, confers tolerance to drought in tobacco [48]. The substrate of GhMKK3 under drought stress is GhMPK7. *GhMKK3* overexpression plants displayed larger stomatal apertures but lower stomatal densities upon ABA treatment-induced stomatal closure, suggesting that GhMKK3-GhMPK7 increases tolerance to drought stress (Figure 1c), which is also related to ABA-mediated stomatal closure. In addition, GhMPK7 interacts with GhPIP1 [48], which is a plasma membrane intrinsic protein involved in water stress [80] (Figure 1c). GhPIP1 is likely the substrate of GhMPK7. Additional experimental studies should elucidate whether GhPIP1 is the substrate of GhMPK7 under drought stress. It has also been reported that GhMPK2 regulates drought stress via ABA-mediated stomatal closure [52]. In rice, OsMKK10.2-OsMPK3 has been implicated in conferring tolerance to drought stress via ABA signaling [11]. After drought treatment, *OsMKK10.2* overexpression plants showed a higher survival rate than WT plants, whereas *OsMKK10.2*-RNAi plants displayed a lower survival rate, suggesting that OsMKK10.2 positively regulates drought stress. When *OsMKK10.2* was overexpressed in *OsMPK3*-RNAi mutant plants, the phenotype of drought tolerance disappeared, suggesting that OsMPK3 acts downstream of OsMKK10.2 under drought conditions. Moreover, the phosphorylation of OsMPK3 was decreased in an *osphs3* mutant (ABA-deficient mutant), suggesting that OsMKK10.2-OsMPK3 increases tolerance to drought stress via ABA signaling [11]. However, the direct evidence linking the MAPK cascade with ABA signaling is unclear. Recently, some research proved MAPKKK can directly interact with ABA signaling modules. ABI (PP2C ABA Insensitive2) dephosphorylates AtMAPKKK18 without ABA treatment and induces AtMAPKKK18 degradation. When plants perceive ABA, ABI1 interacts with PYR (PYRABACTIN Resisitance1)/PYL (PYR-Like) receptors and AtMAPKKK18 becomes stabilized [81]. It was proved that ABA signaling modules directly regulate MAPKKK. However, there is no idea about how AtMAPKKK18 is activated under drought stress. Up to 2020, three studies have proved RAFs (Raf like kinases) can directly phosphorylate SnRK2s under drought/mimic drought conditions [47,82,83]. AtM3Kδ1 phosphorylates OST1/ SnRK2.6 via ABA-induced stomatal closure [82]. AtRAF18, AtRAF20 and AtRAF24 phosphorylate and activate the subclass I SnRK2 kinases SRK2A/SnRK2.4 and SRK2G/SnRK2.1 under drought stress [47]. B4 subfamily RAFs activate SnRK2.1/4/5/9/10 and B2/3 subfamily RAFs activate SnRK2.2/3/6 under ABA-mediated PEG treatment [83]. Upon these findings, it is likely that RAFs activate SnRK2s and, in turn, MAPKKK18, eventually conferring tolerance to drought. This speculation needs more experimental evidence to be proven. Furthermore, RAFs and SnRK2s localize at the cytoplasm and nucleus, respectively. The upstream sensors that activate RAFs need to be verified.

ROS scavenging is another major mechanism regulating drought stress. Upregulating the expression of antioxidative genes and increasing enzyme activities are major ways to scavenge ROS. *OsDSM1* is a Raf-like MAPKKK that enhances tolerance to drought stress in rice by increasing POX activity [37]. *PtMKK4* enhanced tolerance to drought stress in poplar by stimulating SOD and POD activities [49]. The overexpression of *GhMKK1* in tobacco increased tolerance to drought stress by increasing POD, CAT, SOD and APX activities, especially POD activity [51]. Overexpressing *GbMPK3* in tobacco induced the transcription levels of *NbAPX*, *NbCAT* and *NbGST* in transgenic plants [53]. Overexpressing *BdMKK6*.*2* in tobacco upregulated the expression of *NtRbohD* and *NtRbohf*, which produced ROS in tobacco cells, eventually reducing tolerance to drought [58]. Based on these studies, maintaining ROS homeostasis is a key biological process by which plants balance drought stress and survival. The molecular mechanisms by which MAPK regulates antioxidative enzyme activities to control ROS production under drought stress need to be clarified.

As common substrates of MAPK, WRKY transcription factors can bind the promoters of drought stress response genes to regulate their expression, eventually controlling drought stress. An integrated MAPK cascade comprising the GhMAP3K15, GhMKK4 and GhMPK6 modules plays a key role in regulating drought stress in cotton [45] (Figure 1c). Virus-induced gene silencing (VIGS) of *GhMAP3K15*, *GhMKK4* and *GhMPK6* decreased tolerance to drought in cotton. Protein interaction and phosphorylation assays further proved that GhMAP3K15 can phosphorylate GhMKK4, which, in turn, phosphorylates GhMPK6. Moreover, GhWRKY59 was identified as a substrate of GhMPK6 under drought treatment. GhWRKY59 has two major biofunctions in regulating drought stress. On the one hand, GhWRKY59 can bind the promoter of *GhDREB2* and activate the expression of *GhDREB2*, which positively regulates drought stress. On the other hand, GhWRKY59 controls *GhMAP3K15* expression; hence, a positive feedback loop exists between GhWRKY59 and GhMAP3K15 [45] (Figure 1c). Another complete MAPK cascade comprising GhMAP3K14, GhMKK11 and GhMPK31 is also involved in drought stress, but its function under drought stress requires additional genetic and molecular evidence [44]. OsWRKY30 is a positive regulator of drought stress. OsMPK3, OsMPK7 and OsMPK14 phosphorylate OsWRKY30 in vitro, suggesting that OsMPK3/7/14 may be upstream of OsWRKY30 under drought stress [84]. Additional genetic evidence is needed to determine whether OsMPK3, OsMPK7 and OsMPK14 are upstream of OsWRKY30 under drought stress, requiring additional phosphorylation analyses. Moreover, whether OsMPK3, OsMPK7 and OsMPK14 perform redundant functions in drought stress is unclear. To date, the substrate of the MAPK cascade under drought stress has been identified, but the upstream MAPK cascade in drought stress remains unknown. Further studies should exert efforts to identify the RLKs and receptor-like proteins (RLPs) upstream of MAP3K and their function in drought stress.

## 4. MAPK in Temperature Stress

Due to global warming, the frequency of extreme weather has already increased, especially during the winter and summer [5]. Temperature stress induces a broad spectrum of physiological processes and molecular mechanisms. [85,86]. To survive, plants need to adjust at the cellular, metabolic and molecular levels to increase tolerance to temperature stress [87]. As a major signal transducer, the MAPK cascade regulates plant resistance to temperature stress by phosphorylating downstream substrates to directly modify temperature-related gene expression and changing cellular metabolism (increasing compatible solutes and antioxidative enzyme activities).

**Figure 2:**
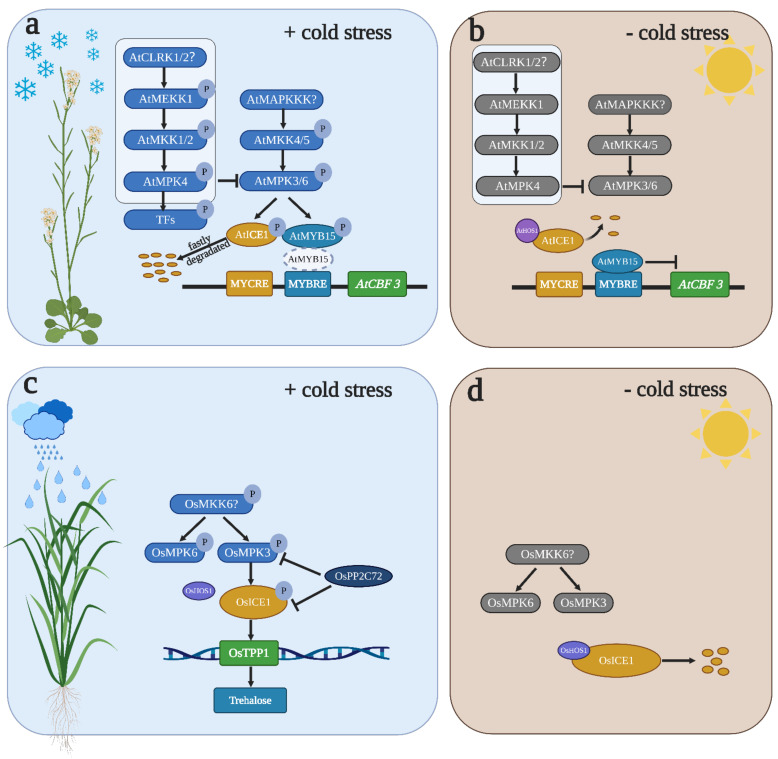
MAPK cascade regulates cold stress in *Arabidopsis* and rice. (**a**,**b**) In *Arabidopsis*, two pathways can regulate cold stress. AtMEKK1-AtMKK2-AtMPK4 positively regulates cold stress, whereas AtMKK4/5-AtMPK3/6 negatively regulates cold stress. (**a**) When cold stress occurs, AtMPK3/6 can phosphorylate AtICE1 and AtMYB15, which induces AtICE1 fast degradation and represses AtMYB15’s binding affinity, which, in turn, attenuates *AtCBF3* transcription. The upstream targets of AtMKK4/5 are unknown (marked as ?). AtMEKK1-AtMKK2-AtMPK4 suppresses AtMPK3/6 activity. The substrate of AtMPK4 is still unknown (named TFs). (**b**) In the absence of cold stress, AtMPK3/6 cannot phosphorylate AtICE1 and AtMYB15. AtICE1 can be degraded by 26S proteasome, and AtMYB15 can bind the promoter of *AtCBF3* to suppress *AtCBF3* expression. (**c**,**d**) The OsMPK3-OsICE1 cascade regulates cold stress in rice. (**c**) Upon cold stress treatment, OsMPK3 phosphorylates OsICE1, which represses the interaction between OsICE1 and OsHOS1 and eventually induces *OsTPP1* expression and trehalose production. OsMKK6 and other MAPKKs (Mitogen-activated protein kinase kinase) (marked as ?) are shown as upstream positive or negative regulators of this MAPK cascade. OsPP2C72 can dephosphorylate OsMPK3 and OsICE1 which represses the function of OsMPK3 and OsICE1 under cold stress. (**d**) Under warm temperature, OsMPK3 cannot phosphorylate OsICE1, which can be degraded by OsHOS1. This figure was created using BioRender (http://biorender.com/; accessed on 14 December 2020).

An MAPK cascade consisting of AtMEKK1-AtMKK1/2-AtMPK4/6 has been implicated in the positive regulation of cold stress in *Arabidopsis* [32,88]. It has been reported that *atmkk2* single-mutant plants displayed reduced tolerance to cold stress [32]. Recently, it has been shown that compared to WT plants, *atmkk2* single mutants did not show any sensitivity to freezing. The expression of *AtCBF* genes did not differ in *atmkk1* or *atmkk2* single mutants, but the *AtCBF* genes were slightly upregulated in the *atmkk1 atmkk2* double mutants, suggesting that AtMKK1 and AtMKK2 perform redundant functions in controlling cold stress [88]. A previous study indicated that cold stress can induce Ca^2+^ accumulation in cells [89,90]. However, Ca^2+^-mediated MAPK signal transduction is still unclear. Ca^2+^ accumulation can be sensed by RLKs which localize in the membrane. It has been reported that AtCLRK1, which is a Ca^2+^/CaM-associated RLK, can regulate cold stress by interacting with and phosphorylating AtMEKK1 [91,92]. AtCLRK1 is possibly a sensor that senses Ca^2+^ accumulation after cold treatment and induces AtMEKK1 activation. Recently, it has been shown that AtCLRK1 and AtCLRK2 perform redundant functions in cold stress which positively regulate cold stress [88] (Figure 2a), although AtCLRK1/2 act as a sensor of Ca2+/CaM in cold stress and trigger MAPK cascade activation. Additional reverse genetic analyses are needed to verify the relationship between AtCLRK1/2 and the AtMEKK1-AtMKK1/2-AtMPK4/6 cascade. In contrast, another MAPK cascade comprising AtMKK4/5 and AtMPK3/6 negatively regulates cold stress in *Arabidopsis* [88]. Although AtMPK6 may increase tolerance to cold stress [32], direct evidence of how AtMPK6 regulates cold stress is lacking. It has been shown that AtMPK3 and AtMPK6 play a strong antagonistic role with AtMPK4 in cold stress in *Arabidopsis* [88]. Freezing tolerances are observed in *atmpk3* and *atmpk6* single mutants and MPK6SR (*atmpk3*/*atmpk6* double mutant), whereas *AtMKK5^DD^*-induced plants exhibit decreased cold tolerance. The kinase activities of AtMPK3 and AtMPK6 are activated in *AtMKK5^DD^*-induced plants but not in the *atmkk1/2* mutant, suggesting that AtMKK4/5 are upstream of AtMPK3/6 but not AtMKK1/2. Freezing sensitivity mediated by the AtMKK4/5-AtMPK3/6 cascade is related to the changeable expression of *AtCBF* genes; *AtCBF1*, *AtCBF2* and *AtCBF3* are significantly upregulated in *atmpk6* and MPK6SR mutants but significantly downregulated in *AtMKK5^DD^*-induced plants [8,88]. A previous study showed that AtYDA is upstream of AtMKK4/5-AtMPK3/6 in stomatal development [93], but AtYDA is not the upstream AtMAP3K of AtMKK4/5-AtMPK3/6 in the cold response [88] (Figure 2a). Further studies should be performed to identify which AtMAP3K is involved in AtMPK3/6-mediated cold sensitivity and verify the relationship between this AtMAP3K and AtYDA because the transcripts of three *AtCBF* genes accumulate in the *atyda* mutant after cold treatment, and they also need to verify the mechanisms of MPK4 when suppressing the activity of AtMPK3/6.

The critical roles of MPK3, MPK4 and MPK6 in cold stress are well known [8,32,88]. However, the biological function of MPK3, MPK4 and MPK6 in freezing responses is still unclear. Hence, identifying the specific substrates of MPK3, MPK4 and MPK6 is a direct way to discover the exact function of MPK3, MPK4 and MPK6 in freezing responses. To date, multiple genetic and biochemical studies have already elucidated that AtMYB15, AtICE1, SlSPRH1 and OsbHLH002 are substrates of MPK3 and MPK6 under temperature stress in *Arabidopsis*, rice and tomato [8,9,88,94,95] (Figure 2). However, to date, the specific substrate of MPK4 under cold stress has not been identified (Figure 2a). Previous studies have already clarified that the ICE1-CBF-COR module plays a key role in cold acclimation [96,97] (Figure 2b). Uncovering the upstream signal affecting ICE1 stability and transcriptional activity is important for controlling cold tolerance. In *Arabidopsis*, AtMPK3 and AtMPK6, which are the upstream kinases of AtICE1, phosphorylate AtICE1 and promote AtICE1 degradation [8,88] (Figure 2a). The expression of *AtCBF* genes did not obviously differ between *atmpk3/atice1* and *atmpk6*/*atice1* double mutants, but the expression of *AtCBF* genes was rescued in *AtMKK5^DD^/ pro AtICE1: AtICE1-YFP* plants. This genetic evidence fully supports that AtICE1 is epistatic to AtMPK3/6 in genetic position. Protein interaction and phosphorylation assays prove that AtMPK3 and AtMPK6 interact and phosphorylate AtICE1. Such phosphorylation achieves dual-level regulation of AtICE1. On the one hand, AtMPK3- and AtMPK6-mediated phosphorylation affects AtICE1 transcriptional activity, which, in turn, attenuates the ability to bind the *AtCBF3* promoter. When the phosphorylation sites of AtICE1 are mutated to an inactive (AtICE1^6A^) and phosphor-mimic status (AtICE1^6D^), transgenic plants show opposite phenotypes after chilling treatment. *AtICE1^6A^/atice1*, but not *AtICE1^6D^/atice1*, rescued the freezing sensitivity of *atice1*, suggesting that the function of AtICE1 in freezing stress is repressed after phosphorylation. Moreover, the transcriptional activities of *GUS* were reduced in AtICE1^WT^ and AtICE1^6D^ but enhanced in AtICE1^6A^ when *AtICE1* and *proCBF3*::*GUS* were co-transformed in tobacco. On the other hand, AtMPK3- and AtMPK6-mediated phosphorylation affects AtICE1 stability. The protein level of AtICE1 is reduced in *AtICE1^6D^/atice1* and *AtICE1/atice1* mutants but obviously increased in *AtICE1^6A^/atice1* mutants, suggesting that phosphorylation promotes the ubiquitination of AtICE1 and ultimately promotes AtICE1 degradation [8,88] (Figure 2a). Furthermore, it has been shown that OsMPK3 can also phosphorylate OsICE1 (OsbHLH002) in rice, but the influence of phosphorylation is opposite in rice. Under warm conditions, OsHOS1 interacts with OsICE1 and induces OsICE1 degradation (Figure 2d). However, upon cold stress, OsMPK3 can phosphorylate OsICE1, enhance *OsICE1* transcriptional activity and promote OsICE1 stability by inhibiting OsHOS1-mediated OsICE1 ubiquitination [95] (Figure 2c). A previous study showed that OsMKK6 can activate OsMPK3 and OsMPK6 to increase resistance to chilling stress in rice [98] (Figure 2c,d). Thus, OsMKK6 may be upstream of OsMPK3-OsICE1-OsTPP1, but additional experimental evidence is needed to prove this hypothesis. Recently, it was shown that OsPP2C72 can interact with OsMPK3 and OsICE1 in planta. More importantly, OsPP2C72 can directly dephosphorylate OsMPK3 and OsICE1 to prevent the positive effect of the OsMPK3-OsICE1-OsTPP1 module under cold stress [99] (Figure 2d). In addition, another transcriptional network composed of AtMPK6-AtMYB15-AtCBF-AtCOR plays a key role in cold stress in *Arabidopsis* [94] (Figure 2c). AtMYB15 is a repressor of AtCBF that can bind to the *AtCBF* promoter and inhibit *AtCBF* expression [100] (Figure 2b). AtMPK6 can phosphorylate AtMYB15 at the Ser 168 residue. When Ser 168 was mutated to Ala, the binding affinity of AtMYB15^S168A^ was significantly increased in the presence of AtMPK6 and ATP, but the binding affinity of AtMYB15^WT^ was almost abrogated. In addition, *AtMYB15^S168A^*-OX (overexpressing) plants are more sensitive to chilling stress than *AtMYB165^WT^*-OX and WT plants, suggesting that AtMPK6-mediated AtMYB15 phosphorylation reduces the binding affinity to AtCBF and enhances the *AtCBF* transcription levels, ultimately conferring tolerance to cold [94] (Figure 2a). Overall, the direct connection between MAPK and CBF genes has been revealed. However, some important questions remain to be answered. First, the relationship between AtICE1 and AtMYB15 needs to be verified because AtICE1 and AtMYB15 are substrates of AtMPK6 but play an antagonistic role in regulating *AtCBF* expression. Second, whether OsTPP1 can regulate the OsCBF-OsCOR gene expression cascade is unknown because OsTPP1 can be phosphorylated by OsICE1, and OsTPP1 positively regulates chilling stress. The substrate of MPK6 under high-temperature (HT) stress has been identified in tomato. SlMPK1 is an ortholog of AtMPK6 in tomato. SlSPRH1 is a substrate of SlMPK1. SlMPK1 and SlSPRH1 are negative regulators of HT stress [9]. Further studies should focus on identifying the substrate of SlSPRH1 to deeply understand the molecular mechanism by which SlMPK1-SlSPRH1 mediates HT stress sensitivity.

In addition to the molecular reactions that change under temperature stress, some physiological processes are already changed in adaptation to adverse temperature factors. The MAPK cascade regulates temperature stress by changing compatible solute contents and antioxidative enzyme activities. Soluble sugar, proline, MDA and REL contents are four major physiological parameters of abiotic stress [101,102]. Antioxidative enzyme activities reflect ROS scavenging ability, which maintains oxidative homeostasis under temperature stress. Overexpressing *ZmMKK1* in tobacco confers chilling tolerance to plants by accumulating soluble sugars and proline and decreasing the MDA and REL levels after chilling treatment. The activities of POD, SOD, CAT and APX are significantly increased after chilling treatment in *ZmMKK1*-overexpressing transgenic plants [103]. Overexpressing *ZmMPK1* in *Arabidopsis* enhances tolerance to heat stress by increasing the proline contents and decreasing the MDA contents [104]. Furthermore, it has been shown that the overexpression of *SlMPK7* and *SlMPK3* confers tolerance to chilling stress in tomato [105,106]. Under chilling stress, the MDA and REL contents are reduced in overexpression plants, but the soluble sugar and proline contents obviously accumulate in transgenic overexpression plants. Moreover, POD, SOD and CAT activities are increased in transgenic overexpression plants under chilling stress [105,106]. Although these studies broaden our understanding of the MAPK function under temperature stress in vegetable plants, the complete MAPK cascade needs to be identified to reveal the molecular mechanisms underlying temperature stress in vegetable plants.

## 5. MAPK in Biotic Stress

Potential pathogens exist in the air and soil and consistently threaten plant adaption and crop productivity [12]. Using chemical pesticides in planting areas is the most common strategy, but this method dramatically destroys the balance between humans and ecology. Cultivating resistant crops has become the most effective and environmentally friendly way to address this serious problem [107]. During a long period of plant–pathogen interactions, plants have evolved sophisticated immune systems to prevent pathogens from invading. [108]. The MAPK cascade plays a critical role in the plant defense response. MPK3, MPK4 and MPK6 are activated after pathogen perception to induce an early defense response [109]. MPK3, MPK4 and MPK6 regulate plant disease resistance by regulating phytoalexin and phytohormone biosynthesis in biotic stress and activating downstream substrates, which play a vital role in the early plant defense response. The upstream of MPK3, MPK4 and MPK6 in biotic stress has already been identified [110,111,112,113]. Other studies also showed the function of other MAPK cascade members in biotic stress.

**Figure 3:**
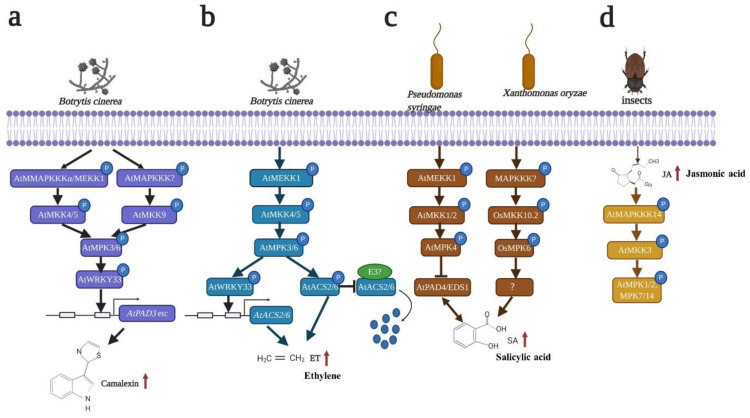
MAPK cascade function in biotic stress. (**a**) The MAPK cascade is involved in camalexin biosynthesis after *Botrytis cinerea* infection. (**b**) The AtMEKK1-AtMKK4/5-AtMPK3/6 cascade regulates ethylene (ET) production in two dependent ways. In one approach, AtMPK3/6 phosphorylates AtWRKY33, which can bind the promoters of *AtACS2* and *AtACS6* and activate *AtACS2* and *AtACS6* expression. In the other approach, AtMPK3/6 can directly phosphorylate AtACS2 and AtACS6, eventually promoting AtACS2 and AtACS6 stability. (**c**) AtMEKK1-AtMKK1/2-AtMPK4 negatively regulates salicylic acid (SA) production by negatively regulating AtPAD4 and AtEDS1 activities, whereas OsMKK10.2-OsMPK6 positively regulates SA production. (**d**) AtMAPKKK14-AtMKK3-AtMPK1/2/7/1 can be activated by jasmonic acid (JA) after insect feeding. The red arrows mean content increase. This figure was created using BioRender (http://biorender.com/; accessed on 14 December 2020).

When pathogens enter plants, the plants produce phytoalexins in response and initiate disease resistance [114,115]. Phytoalexins are low-molecular weight antimicrobial compounds that differ among species [110]. As a major phytoalexin, camalexin (3-thiazol-2-yl-indole) accumulates after *Botrytis cinerea* and other pathogen infections [63,116]. It has been reported that AtMPK3 and AtMPK6 play key roles in camalexin production in *Arabidopsis* [63,117,118] (Figure 3a). There are two major ways to activate AtMPK3 and AtMPK6, which finally induce camalexin production. One way is AtMAPKKKα/AtMEKK1-AtMKK4/AtMKK5-mediated AtMPK3 and AtMPK6 activation [63,117]. The production of camalexin is reduced in *atmpk3* single mutants but delayed in *mpk6* single mutants. Moreover, the expression of *AtPAD3* is almost reduced and delayed in *atmpk3* and *atmpk6* mutants, suggesting that AtMPK3 and AtMPK6 perform redundant functions in *B. cinerea*-induced camalexin accumulation. Gain-of-function genetic and epistatic analyses have revealed that AtMAPKKKα/AtMEKK1 and AtMKK4/AtMKK5 are upstream of AtMPK3 and AtMPK6 and are necessary for AtMPK3 and AtMPK6 activation [63] (Figure 3a). Another way is AtMKK9-induced AtMPK3 and AtMPK6 activation [118] (Figure 3a). *AtMKK9^DD^* transgenic plants accumulate more camalexin than *AtMKK4^DD^* and *AtMKK5^DD^* transgenic plants after Dex induction in *Arabidopsis*, suggesting that AtMKK9, AtMKK4 and AtMKK5 perform overlapping functions in camalexin production. AtCYP79B2 and AtCYP79B3 are two major enzymes that catalyze Trp conversion to indole-3-acetaldoxime (IAOx). AtCYP71A13 and AtPAD3 are critical for camalexin biosynthesis [119]. The transcription levels of *AtCYP79B2*, *AtCYP79B3*, *AtCYP71A13* and *AtPAD3* are strongly induced in *AtMKK9^DD^* plants but partially compromised in *AtMKK9^DD^/atmpk3* and *AtMKK9^DD^/atmpk6* plants, suggesting that AtMKK9 is essential for *MPK3* and *AtMPK6* activation [118] (Figure 3a). However, the relationship between AtMKK9 and AtMKK4/5 in camalexin production is still unclear. An *atmkk4/5/9* triple mutant is needed for further research.

A substrate of AtMPK3 and AtMPK6, i.e., AtWRKY33, regulates camalexin biosynthesis in four different ways. First, AtWRKY33 directly binds the *AtPAD3* promoter and activates *AtPAD3* expression, which is involved in camalexin biosynthesis [117,120] (Figure 3a). Second, AtWRKY33 can bind its own promoter, which activates *AtWRKY33* expression and eventually activates *AtPAD3* expression [117]. Third, AtWRKY33, AtMPK4 and AtMKS1 naturally form a complex in the nucleus. After *Pst DC3000* (*Pseudomonas syringae* pv. *maculicola*) infection or flg22 treatment, AtWRKY33 is released from this complex and binds the promoter of *AtPAD3* to regulate *AtPAD3* expression [120]. Fourth, AtWRKY33 can bind multiple genes in the camalexin biosynthesis process according to ChIP-seq analyses [121] (Figure 3a). Based on these findings, the transcription factor AtWRKY33 is critical for camalexin production. However, some questions remain unsolved. First, AtMPK3 and AtMPK6 can phosphorylate AtWRKY33, and AtMPK4 can form a complex with AtWRKY33. The relationship among AtMPK3, AtMPK6 and AtMPK4 needs to be verified. Second, additional experimental evidence is needed to further determine whether AtWRKY33 can directly regulate multiple genes in addition to *AtPAD3* in the camalexin biosynthesis process.

In addition to camalexin, other phytoalexins can be induced by the MAPK cascade. The OsMKK4-OsMPK6 pathway specifically activates numerous genes involved in diterpenoid phytoalexin biosynthesis, thereby regulating diterpenoid phytoalexin biosynthesis [122]. NtSIPK (salicylic acid-induced protein kinase) and NtWIPK (wound-induced protein kinase) phosphorylate NtWRKY8, and NtWRKY8 upregulates the expression of *HMGR2* (gene encoding 3-hydroxy-3-methylglutaryl CoA reductase 2), which is the rate-limiting enzyme in isoprenoid production in tobacco [123]. AtMPK3 also induces the accumulation of the phytoalexin scopoletin. However, how MAPK regulates diterpenoid and scopoletin phytoalexin production requires further investigation.

Salicylic acid (SA), JA and ET are three major plant hormones involved in the plant defense response [112,124,125]. Several studies have reported that the MAPK cascade participates in JA, SA and ET biosynthesis and signaling [126,127].

The MAPK module plays a key role in ET biosynthesis and signaling. The MAPK cascade controls ET biosynthesis by regulating the rate-limiting step of ET biosynthesis. ACS is the rate-limiting enzyme in ET biosynthesis [128]. In *Arabidopsis*, AtMPK3 and AtMPK6 regulate AtACS2 and AtACS6 at the transcriptional, posttranscriptional and protein stability levels. On the one hand, AtMPK3 and AtMPK6 regulate *AtACS2* and *AtACS6* expression [129]. AtMPK3 and AtMPK6 phosphorylate AtWRKY33, which binds the promoters of *AtACS2* and *AtAC*S6 and activates *AtACS2* and *AtACS6* expression, eventually resulting in cellular ET accumulation [117,129] (Figure 3b). On the other hand, AtMPK3 and AtMPK6 can directly phosphorylate AtACS2 and AtACS6, which enhances AtACS2 and AtACS6 protein stability. AtACS2 and AtACS6 can be degraded by the ubiquitin-proteasome pathway, whereas AtMPK3 and AtMPK6 phosphorylate AtACS6, which reduces AtACS6 degradation, eventually promoting AtACS6 stability and inducing ET production [15,17,130] (Figure 3b). AtMKK4 and AtMKK5 are upstream of AtMPK3 and AtMPK6, which perform redundant functions in ET production in *Arabidopsis* [15,130] (Figure 3b). In addition to AtMKK4 and AtMKK5, it has also been shown that ZmMKK10 positively regulates ET biosynthesis [131]. ZmMKK10 exhibits 46.8% similarity to AtMKK9. *ZmMKK10^DD^*-overexpressing plants induced ethylene accumulation under normal conditions. AVG (Aminoethoxy vinyl glycine) and CoCl_2_ are inhibitors of ACSs and ACOs, respectively. *ZmMKK10^DD^*-overexpressing plants treated with AVG and CoCl_2_ display reduced ET production. Reverse genetic and epistatic analyses further proved that ZmMPK3 and ZmMPK7 are substrates of ZmMKK10 in ET production [131]. Further studies should elucidate whether ZmMKK10, AtMKK4 and AtMKK5 perform redundant functions in ET production.

Ethylene signaling pathways have already been well studied [132,133]. The MAPK cascade regulates ET signaling through two independent pathways. On the one hand, AtMPK6 phosphorylates AtERF6, which activates *AtPDF1.2a* and *AtPDF1.2b* expression. AtMPK6 phosphorylates AtERF6 at the Ser 266 and Ser 269 residues. Such phosphorylation increases the transcriptional activity of genes that have GCC boxes in their promoter regions [134]. In addition, AtMPK6 phosphorylates AtERF104, which can bind GCC box *cis*-elements, i.e., the potential target genes of AtERF104, including *AtPDF1.2* and *AtPDF1.2b*, which can be significantly upregulated in *AtERF104* overexpression plants [135].

SA also plays a critical role in the plant defense response and can be regulated by the MAPK cascade [126]. MPK3, MPK4 and MPK6 are involved in regulating SA biosynthesis and signaling. *CA-MPK3 (*Constitutively active *AtMPK3)* in *Arabidopsis* increased the SA levels, but overexpressed *CA-AtMPK3* on an *atsid2* background impaired SA biosynthesis, suggesting that AtMPK3 plays a key role in SA biosynthesis [136] (Figure 3). OsMKK10.2 phosphorylates OsMPK6 after *Xoc* (*Xanthomonas oryzae* pv. *oryzicola*) infection, whereas phosphorylation is impaired in *nahG* transgenic plants (SA-deficient transgenic rice), suggesting that OsMPK6 confers resistance to *Xoc* via SA [11] (Figure 3c). In contrast to MPK3 and MPK6, MPK4 plays a negative role in SA production [137,138]. *The atmekk1* and *atmkk1/2* mutants exhibit accumulated cellular SA levels. However, when *nahG* is expressed in *atmekk1* and *atmpk4* mutant plants, *nahG* rescues the *atmekk1* and *atmpk4* dwarf phenotypes and compromises resistance to pathogens [139,140,141], suggesting that AtMEKK1-AtMKK1/2-AtMPK4 negatively regulates the defense response by reducing the endogenous SA levels [120,140,142,143] (Figure 3c). In addition, AtMKK7 plays a critical role in systemic acquired resistance (SAR) by regulating SA biosynthesis and signaling. The expression of *AtMKK7* is increased in *bud1* mutants (elevated SA mutants), which increases the SA levels and *PR* gene expression. The ectopic expression of *AtMKK7* in local tissues induces SA accumulation and *AtPR1* expression and enhances resistance to *Psm* ES4326 in systemic tissues, indicating that AtMKK7 is necessary for SA-induced SAR [144]. SlMPK3 increases resistance to TYLCV (tomato yellow leaf curl virus) by activating SA signaling, and the expression of *SlPR1* and SlPR1b is increased in *SlMPK3*-overexpressing plants [145]. Further studies should focus on the substrates of MPK3 and MPK6, which can regulate SA-related genes to control SA biosynthesis and signaling.

After pathogen infection, herbivore attacks and mechanical wounding, JA biosynthesis and signaling are activated [125]. The MAPK cascade has been reported to regulate both JA biosynthesis and signaling. In tobacco, NtSIPK and NtWIPK trigger JA accumulation, but NtMEK2^DD^, which is upstream of NtSIPK and NtWIPK, does not increase the JA levels in cells. MKK2 is likely insufficient to induce JA production [146,147]. To date, which MKK is necessary and sufficient to induce JA production has not been determined. In tomato, LeMPK1, LeMPK2 and SlMPK3 are involved in JA production and signaling. After *Manduca sexta* (Lepidoptera) feeding, the overexpression of *LeMPK1* and *LeMPK2* transgenic plants induced JA accumulation, whereas the co-silencing of LeMPK1 and LeMPK2 reduced JA production, suggesting that LeMPK1 and LeMPK2 confer tolerance to herbivorous feeding by accumulating the endogenous JA levels [148]. SlMPK3 enhances resistance to TYLCV inoculation by activating JA signaling, which increases *SlLapA*, *SlPI-I* and *SlPI-II* expression after virus inoculation in overexpressing *SlMPK3* transgenic plants [145]. In *Arabidopsis*, AtMPK4 and AtMPK6 are also involved in JA signaling. In the *atmpk4* mutant, *AtPDF1.2* and *AtTHI 2.1* are not expressed under normal conditions, and even after MeJA treatment, the expression of *AtPDF1.2* and *AtTHI 2.1* could not be detected, suggesting that AtMPK4 may positively regulate the JA signaling pathway [139]. It has also been reported that AtMPK6 along with AtMKK3 negatively regulates *AtMYC2* expression, which can control JA signaling [149]. Recently, another report also showed that JA can activate the MAPK cascade in *Arabidopsis*. AtMAPKKK14- AtMKK3-AtMPK1/2/7/14 can be activated after insect feeding, and their activities are controlled by the JA levels, suggesting that potential feedback may exist between the MAPK cascade and JA [150] (Figure 3d).

The above findings indicate that MAPK cascades regulate plant immunity by phosphorylating specific transcription factors or regulating a specific gene expression. This change seems to be limited. In other words, it is not sufficient for plants to acquire resistance to pathogens. Other mechanisms controlling gene expression in plant immunity need to be identified. Some research has already proved the critical role of histone modification in plant immunity, such as histone acetylation, histone methylation and histone ubiquitination [151]. HUB1 and HUB2 (histone monoubiquitination) are involved in the plant defense response to necrotrophic fungi in *Arabidopsis* and tomato [152,153]. AtSRT2 (HDAC SIRTUIN2), AtHDA19, AtHDA6 and OsHDT701, some histone deacetylases, also regulate plant immunity [154,155,156,157]. Some histone demethylases have been associated with the regulation of the plant defense response, such as AtJMJ27, OsJMJ705 and OsJMJ704 [158,159,160]. More importantly, AtMKK5 and AtMKK3 loci displayed increased H3K36me3 and decreased H3K36me1 in the *Col-0* but not in the *sdg8* mutant (histone methyltransferase SET DOMAIN GROUP8) in response to infection. Therefore, histone methylation can directly change the expression of MKK3 and MKK5 at early signaling of the defense response [161]. However, whether MAPK cascades can regulate the plant defense response by global chromatin reprogramming remains unclear. Recently, it has been reported that MAPK cascades can regulate plant immunity by involvement in histone acetylation. MPK3 acts as a key regulator in histone modification-mediated chromatin modulation in microbe-associated molecular pattern (MAMP)-triggered plant immunity. MPK3 phosphorylates HD2B (a histone deacetylase) in vivo. HD2B localizes from the nucleolus to the nucleoplasm, where it removes H3K9ac marks in several loci, thereby leading to a global change in defense gene expression [162]. This finding fills the gap between MAPK cascades and global gene expressions after pathogen perception. It provides new cues to explain that MAPK cascades are a key regulator for plant immunity. However, some central questions still need to be resolved, such as the question of whether MAPK cascades also directly regulate histone methylation and histone ubiquitination. Further study needs to pay attention to discovering the role of MPK4 and MPK6 in global changes of gene expression in plant immunity.

## 6. Conclusions

After cells sense environmental stimuli, the MAPK cascade is activated to transform extracellular signaling into intracellular responses. Based on biochemical and genetic analyses and the development of functional genomics, proteomics and phospho-proteomics analyses, information regarding the biofunction of the MAPK cascade under environmental stress has already increased. Additionally, the MAPK network under environmental stresses has become increasingly complex. This phenomenon may be explained by different upstream RLKs/RLPs and specific downstream substrates.

Currently, there are still some challenges in functionally characterizing the MAPK linear pathway. First, the gap between pattern recognition receptors (PRRs) and MAPK in plant immune signaling has already been filled in, but limited success has already existed in understanding the relationship between RLKs/RLPs and MAPK cascades in abiotic stress [163,164]. The reason can be concluded by the large member of RLK and RLP families in plants [12,83,165]. AtMEKK1, AtRAF18, AtRAF20, AtRAF24, RAF40, AtM3Kδ1, AtM3Kδ8, AtM3Kδ7 and AtMAPKKK18 have been well studied in abiotic stress [32,47,82,83,143,166]. AtMEKK1, AtMKKK5, OsMKKK18/24, AtANP1/2 and AtYODA have been identified to function in plant disease resistance [167,168,169,170]. The functions of other MAPKKKs are still unclear. More importantly, RLKs/RLPs function as receptor kinases, and other RLKs/RLPs function as co-receptors and scaffold proteins involved in the receptor complex. Second, the specific signaling transduction of MAPK cascades relies on the docking interaction of MKKs with MAPKs, as well as the specific interaction of MAPKs with substrates [171,172,173]. Moreover, the interaction of MKKs with MAPKs also needs scaffold proteins’ help [174]. However, the mechanisms of the specific interaction between MAPKKKs and MAPKKs are less known [171]. Further study should make efforts to underly this mechanism. Third, abnormal phenotypes are observed in null mutants, especially in plant immunity, and it is difficult to conduct genetic analyses and phenotype analyses. Hence, some new approaches rather than T-DNA insertion, such as chemical strategies, are needed to address these problems [175].

Approximately 20 MAPKs have been identified in *Arabidopsis*, but only three MAPKs (MPK3, MPK4 and MPK6) are well studied in environmental stress. A few advances have revealed the functions of 17 other MAPKs in environmental stress. Hence, the regulatory network of the MAPK cascade seems to be generally single. With the development of high-throughput phospho-proteomics analysis, many transcription factors, enzymes and proteins have been shown to be candidate substrates of MAPKs. Further studies should exert efforts to reveal the function of the 17 other MAPKs in environmental stresses. More importantly, identifying novel substrates of MAPKs is essential to enrich the current understanding of MAPK regulation under environmental stress.

## Figures and Tables

**Table 1:** Summary of mitogen-activated protein kinase (MAPK) genes involved in salt and drought stress responses in Arabidopsis, rice, maize, cotton, etc.

Plant	Gene Name	Stresses	Function in Stresses	References
Arabidopsis	*AtMEKK1*	Salt	Positively regulates salt stress	[32]
	*AtMAP3Kδ4*	Salt	Positively regulates salt stress	[33]
	*AtMAPKKK18*	Drought	Positively regulates drought stress	[34]
	*AtMKK2*	Salt	Positively regulates salt stress	[32]
	*AtMKK3*	Drought	Positively regulates drought stress	[34]
	*AtMKK4*	Salt	Positively regulates salt stress	[35]
	*AtMKK5*	Salt	Positively regulates salt stress	[36]
	*AtMPK3*	Salt	Positively regulates salt stress	[35]
	*AtMPK4*, *AtMPK6*	Salt	Positively regulate salt stress	[32]
Rice	*OsDSM1*	Drought	Positively regulates drought stress	[37]
	*OsMKK10.2*, *OsMPK3*	Drought	Positively regulate drought stress	[11]
Maize	*ZmMAPKKK56*, *ZmMAPKKK19*, *ZmMAPKKK18*	Drought	Induced by drought stress	[38]
	*ZmMKK4*	Salt	Positively regulates salt stress	[39]
	*ZmMKK10-2*	Drought	Positively regulates drought stress	[38]
	*ZmSIMK1*	Salt	Positively regulates salt stress	[40]
	*ZmMPK3*	Drought	Positively regulates drought stress	[41]
	*ZmMPK5*	Salt	Positively regulates salt stress	[42]
	*ZmMPK15*	Drought	Positively regulates drought stress	[38]
	*ZmMPK17*	Salt	Positively regulates salt stress	[43]
Cotton	*GhMAP3K14*	Drought	Positively regulates drought stress	[44]
	*GhMAP3K15*	Drought	Positively regulates drought stress	[45]
	*GhMAP3K6*, *GhMAP3K49*, *GhMAP3K71*, *GhMAP3K92*, *GhMAP3K164*, *GhMAP3K168*	Drought	Induced by drought stress	[44]
	*GhRaf19*	Salt	Negatively regulates salt stress	[46]
	*GhMKK1*	Drought	Positively regulates drought stress	[47]
	*GhMKK3*	Drought	Positively regulates drought stress	[48]
	*GhMKK4*	Drought	Positively regulates drought stress	[49]
	*GhMKK5*	Salt	Negatively regulates salt stress	[50]
	*GhMKK11*	Drought t	Positively regulates drought stress	[51]
	*GhMPK2*	Salt	Positively regulates salt stress	[52]
	*GbMPK3*	Drought	Positively regulates drought stress	[53]
	*GhMPK4*	Salt	Negatively regulates salt stress	[54]
	*GhMPK6*	Drought	Positively regulates drought stress	[45]
	*GhMPK7*	Drought	Positively regulates drought stress	[48]
	*GhMPK17*	Salt	Positively regulates salt stress	[55]
	*GhMPK31*	Drought	Positively regulates drought stress	[44]
Wheat	*TaRaf87*, *TaRaf105*, *TaRaf44*, *TaRaf72*, *TaRaf80*, *TaMKKK16*,	Drought	Induced by drought stress	[56]
	*TaMKK1*	Drought	Induced by drought stress	[56]
	*TaMPK8*	Drought	Induced by drought stress	[57]
*Brachypodium distachyon*	*BdMKK6*.*2*	Drought	Negatively regulates drought stress	[58]

## Data Availability

Not applicable.
